# The complex relationship between oligoclonal bands, lymphocytes in the cerebrospinal fluid, and immunoglobulin G antibodies in multiple sclerosis: Indication of serum contribution

**DOI:** 10.1371/journal.pone.0186842

**Published:** 2017-10-23

**Authors:** Cheryl Beseler, Timothy Vollmer, Michael Graner, Xiaoli Yu

**Affiliations:** 1 Department of Neurosurgery, University of Colorado Denver School of Medicine, Aurora, Colorado, United States of America; 2 Department of Neurology, University of Colorado Denver School of Medicine, Aurora, Colorado, United States of America; 3 yuScience, LLC, BioScience Park Center, Aurora, Colorado, United States of America; Universite Claude Bernard Lyon 1, FRANCE

## Abstract

**Introduction:**

Intrathecal immunoglobulin G (IgG) and oligoclonal bands (OCBs) are the most consistent and characteristic features of Multiple Sclerosis (MS). OCBs in MS are considered products of clonally expanded B cells in the cerebrospinal fluid (CSF), representing the sum of contributions from B cells in the brain. However, large amounts of IgG can be eluted from MS plaques in which lymphocytes are absent, and there is no correlation between levels of plaque-associated IgG and the presence of lymphocytes. It is calculated that it would take 3.2 billion lymphocytes to generate such large amounts of intrathecal IgG (30 mg in 500 ml CSF) in MS patients. Therefore, circulating lymphocytes in CSF could only account for <0.1% of the extra IgG in MS.

**Methods:**

We analyzed clinical laboratory parameters from sera and CSF of 115 patients including 91 patients with MS and 24 patients with other inflammatory central nervous system (CNS) disorders (IC). We investigated the relationship between oligoclonal bands, IgG antibodies, CSF cells, IgG Index, albumin, and total protein.

**Results:**

MS patients have significantly elevated serum concentrations of IgG antibodies, albumin, and total protein, lower levels of lymphocytes, albumin, and total protein in the cerebrospinal fluid, but no difference in CSF IgG concentration compared to those with other inflammatory neurological disorders. Furthermore, in MS there was no linear relationship between the numbers of OCBs, CSF lymphocytes, CSF IgG, and IgG Index, and between serum IgG and serum albumin, but significant correlation between IgG in CSF and serum, and between CSF IgG and CSF albumin.

**Conclusion:**

There are unique differences between MS and patients with other inflammatory neurological disorders. Our data suggest that in MS patient (a) B cells and their products in the CSF may not be the sole source of intrathecal IgG; (b) oligoclonal bands may not be the products of single B cell clones in the CSF; and (c) there is a strong connection between serum components in the peripheral circulation and the central nervous system.

## Introduction

Increased intrathecal synthesis of IgG antibodies and the presence of oligoclonal bands (OCBs) is the most characteristic feature of MS. OCBs are associated with increased levels of disease activity and disability [[1]], a greater risk of second attack [[2]], the conversion from a clinically isolated syndrome (CIS) to early RRMS [[3]], and greater brain atrophy [[4]]. The success of B cell depletion therapies indicates that both B cells and their products may play a critical role in disease pathogenesis.

A distorted self-reactive IgG antibody repertoire was found in MS sera [[5]], and the serum IgG repertoire in the CIS predicts the subsequent development of MS [[6]]. In addition, a pathogenic role of systemic inflammation has been shown to be correlated with disease progression in MS [[7]], and the involvement of antibodies in disease pathogenesis is supported by the clinical response of some patients to treatments known to inhibit antibody-mediated effects in other diseases [[8]]. The potential pathological role of serum antibodies is further supported by evidence that serum antibodies in MS target microvessels in the brain tissues [[9]], disrupt the blood-brain barrier [[10]], and correlate with brain MRI measures of disease severity [[11]]. Furthermore, the drug natalizumab, a peripherally acting anti-VLA4 monoclonal antibody, effectively reduces MS disease activity and decreases CSF IgG levels [[12]]. Natalizumab is an anti-integrin drug that diminishes T cell trafficking in the brain which may explain its clinical efficacy, with the decreased CSF IgG levels possibly being a secondary effect.The finding that B-cell clusters corresponding to OCBs in MS are observed only in the blood [[13]], supports the role of serum IgG antibodies in MS disease pathogenesis. These data suggest a strong connection between IgG in the peripheral blood and that in the CNS of MS patients.

We analyzed serum and CSF clinical lab parameters in 115 patients including 91 MS and 24 IC patients. We present data supporting a complex relationship between OCBs and other CSF parameters, suggesting a strong connection between serum IgG and intrathecal synthesis of IgG in MS.

## Materials and methods

### Patients

All data used for the analyses were extracted from clinical laboratory reports of patients from the University of Colorado Hospital (2001–2013). Serum and CSF samples were collected with approval from the University of Colorado Institutional Review Board (COMIRB # 00–688). Data (Serum and CSF samples) were anonymized before access/analysis. The protein, albumin and IgG concentrations were determined by an independent off-site laboratory using the Siemens BN™ II nephelometry system. The presence of oligoclonal bands was analyzed by ARUP Laboratories (SLC, UT).

### Demographics

Most of the data were from untreated patients. Information on gender was available for 100 patients (76 MS and 24 IC) (71% female). The mean age of MS patients was 42.8, and did not differ significantly from that of IC patients (mean age 49.6). A majority (82 out of 91, 90%) of the MS patients were of the relapsing-remitting type (RRMS). Seven had primary progressive MS, one had the relapsing progressive type, and one was diagnosed as secondary progressive MS. Controls were defined as those with other inflammatory CNS (IC) diagnoses and are listed below with number of cases in parenthesis: acute viral meningitis (2), lymphoma (1), B cell lymphoma (1), Behcet's disease (1), paraneoplastic syndrome (1), viral meningitis (1), Cryptococcal meningitis (1), chronic meningitis of unknown etiology (1), subacute sclerosing panencephalitis (2), acute disseminated encephalomyelitis (1), paraneoplastic encephalitis (1), neurosyphilis, chronic progressive meningoencephalitis (1), sarcoid (1), VZV myelopathy (1), VZV radiculomyelitis (1), VZV shingles (1), ischemic optic neuritis (1), retrobulbar optic neuritis (1), papillitis (1), and transverse myelitis (1).

### Slot blots

Sera and CSF (based on equal IgG concentration) of a serial 3-fold dilutions were blotted in duplicate on nitrocellulose membranes. The blots were incubated in HRP-conjugated goat anti-human IgG (H+L) (Sigma), followed by detection with SuperSignal® West Pico Maximum Sensitivity chemiluminescent substrate (Thermo Scientific). Band intensity analysis was performed using FluorChem Q™ system.

### Statistical analysis

#### Comparison of laboratory parameters

Logarithmic transformations were used to improve normality of serum IgG, CSF IgG, albumin, and protein. Serum albumin and total protein were squared to produce a normally distributed variable. The number of lymphocytes in CSF, OCBs, and the IgG Index could not be transformed to normality. Student’s t-test was used to compare mean differences when variables were normally-distributed. The Wilcoxon non-parametric test were used for the number of CSF cells OCBs. A permutation test for differences in means with 100,000 iterations was used to compare mean levels of total protein in serum, CSF, and IgG Index due to the small sample size in IC patients.

#### Analyses of relationship between OCBs and other CSF parameters

Polynomial regression was applied with a 95% confidence band around the estimated regression lines. We minimized the mean squared error over a number of different linear regression models to identify the maximum level of the predictor where the association changed from a positive to a negative relationship. We then divided the observations into two datasets and conducted significance testing using permutation tests to obtain the p-value for the correlation coefficient, and the slope of the regression lines before and after the cut-off point. A permutation-based p-value was generated because the OCBs were highly skewed and were not normally distributed. Similar analyses were conducted for relationship between OCBs and IgG concentration, and between OCBs and IgG Index.

A sensitivity analysis was conducted to understand the association of number of OCBs and CSF IgG. OCB counts were dichotomized and correlations assessed between each categorical construct and the level of CSF IgG by category.

#### Assessment of relationships between CSF and serum parameters

The Pearson correlation coefficient was used due to the normality of the transformed variables and the linear relationships observed in regression models.

## Results

### MS patients have lower levels of lymphocytes, albumin, and total protein in the CSF, but higher levels of serum IgG and albumin

**Fig 1:**
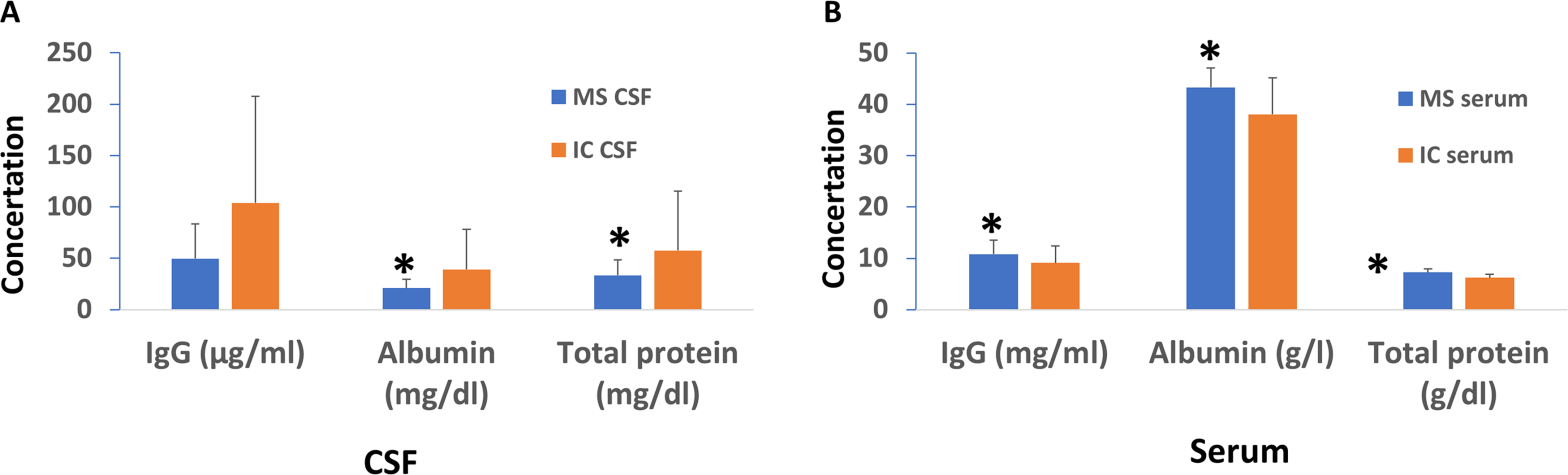
Summary of mean difference in IgG, albumin, and total protein concentrations between MS and IC patients in CSF and serum. MS patients have significantly lower levels of IgG, albumin, and protein concentrations in the CSF (Fig 1A), but significantly higher levels in serum (Fig 1B) compared to IC patients. *p values <0.05.

MS patients had higher incidences of OCBs and higher values of IgG Index, but have no significant difference in CSF IgG levels compared to IC patients. Although CSF IgG in MS was about half the level of IC patients, this difference did not reach significance due to the high variability in CSF IgG concentration in IC patients. The mean differences in the number of CSF lymphocytes in MS patients compared to IC reached statistical significance (Table 1), but with higher number of cells present in IC, consistent with previous reports [[14]]. Interestingly, significantly elevated levels of serum IgG, serum albumin, and serum total protein were found in MS compared to the IC. In contrast, both mean concentrations of CSF albumin and CSF total protein were significantly lower in MS (Table 1). Fig 1 shows the comparison of IgG, albumin, and total protein between MS and IC.

**Table 1:** Comparison of CSF and blood parameters between patients with MS and other inflammatory CNS disorders.

Laboratory Parameter	MS patients Mean ± SD (n)	IC patients Mean ± SD (n)	*p*-value (MS vs. IC)
**Age of patient**	**42.8 ± 10.8 (75)**	**49.6 ± 17.5 (24)**	***p* = 0.08**
**CSF lymphocytes (per μl)**	**6.59 ± 7.12 (61)**	**75.5 ± 19.6 (22)**	***p* = 0.049**
**No. of oligoclonal bands**	**7.02 ± 6.58 (90)**	**3.86 ± 7.11 (22)**	***p =* 0.003**
**CSF IgG (μg/ml)**	**49.7 ± 33.8 (91)**	**103.9 ± 150 (24)**	***p* = 0.29**
**Serum IgG (mg/ml)**	**10.8 ± 2.74 (90)**	**9.11 ± 3.27 (20)**	***P* = 0.03**
**CSF Albumin (mg/dl)**	**20.9 ± 8.55 (70)**	**39.2 ± 29.1 (16)**	***p* = 0.006**
**Serum Albumin (mg/dl)**	**4329 ± 376 (68)**	**3804 ± 715 (16)**	***p* = 0.01**
**IgG Index**	**0.95 ± 0.55 (74)**	**0.55 ± 0.34 (12)**	***p* = 0.003**
**CSF total protein (mg/dl)**	**33.4 ± 15.0 (49)**	**57.6 ± 36.3 (13)**	**p = 0.0007**
**Serum total protein (g/dl)**	**7.25 ± 0.67 (31)**	**6.23 ± 0.69 (6)**	**p = 0.002**

Logarithmic transformations were used to improve normality for CSF IgG, CSF albumin, CSF total protein, and serum IgG. Serum albumin and serum total protein were squared to produce a normally distributed variable. The number of CSF lymphocytes and the number of OCBs could not be transformed to normality. The Student’s t-test was used to compare mean differences when variables were normally-distributed, and the Wilcoxon non-parametric test was used for differences in medians between MS and IC patients for the number of CSF cells and the number of OCBs because they are not normally distributed.

### In MS, the relationships between the number of OCBs and CSF lymphocytes, CSF IgG concentration, and IgG Index are not linear, but demonstrate complex associations

**Fig 2:**
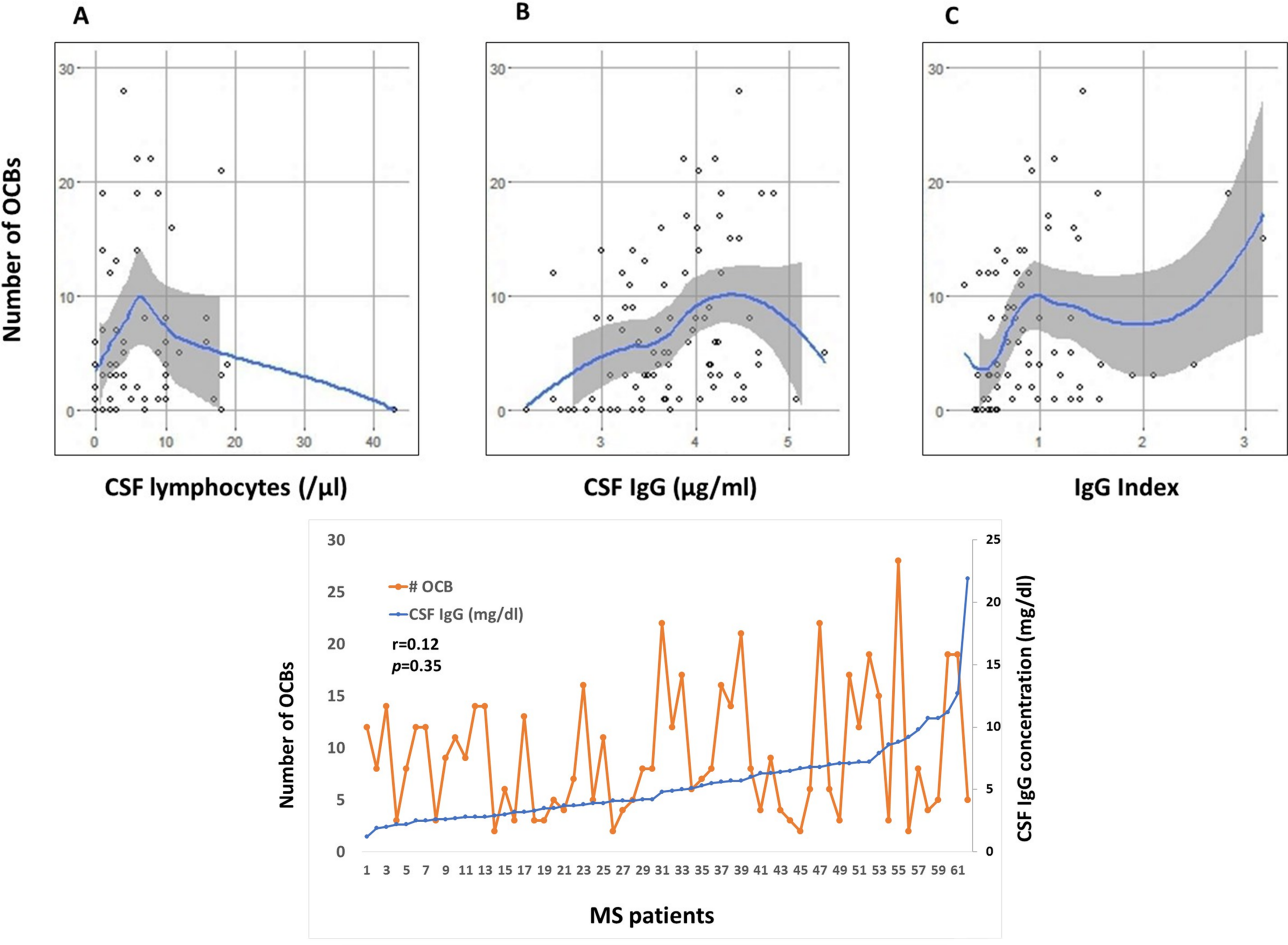
Similar non-linear relationships exist between number of OCBs and CSF lymphocytes, CSF IgG, and IgG Index in MS. A. No linear relationship between number of OCBs and CSF lymphocytes (n = 61) were observed. However, when CSF number of cells = 7, the association changed from an increasing linear association (r = 0.22, p = 0.09) to a decreasing linear association (r = -0.27, p = 0.93). B. No linear relationship between number of OCBs and CSF IgG concentration (n = 63). C. No linear relationship between number of OCBs and IgG Index. However, there is a highly significant positive correlation when IgG Index ≤ 1.14 (r = 0.394, slope = 11.45, p = 0.002), and the correlation becomes decreasing linear association when IgG Index >1.14, although not significant (r = -0.1381, slope = -6.09, p = 0.68). D. No association between CSF IgG concentration and number of OCBs when number of OCBs > 2. Spearman’s correlation coefficient r = 0.12, p = 0.35.

Fitting a polynomial regression curve to the number of CSF lymphocytes predicting the number of OCBs showed a non-linear association between the two parameters. When CSF cells were ≤ 7/μl, a positive, linear association was observed between OCBs and CSF cells (*r* = 0.22, *p* = 0.09, n = 40), and the slope of the regression line was 0.72 (se = 0.52). However, when CSF cells > 7, the linear association was negative (*r* = -0.27, *p* = 0.93, n = 20), and slope of regression line was -0.25 (se = 0.20) (Fig 2A). For CSF IgG, the polynomial fit suggests a linear relationship to OCBs where CSF IgG concentration is less than about 4.72, after which the relationship appears to decrease (n = 84, *r* = 0.34, slope = 3.75, se = 1.13, *p* < 0.0001) (Fig 2B). There were too few data points greater than 4.72 to estimate the regression line at higher levels of CSF IgG. IgG Index is defined as the CSF IgG to CSF albumin ratio compared to the serum IgG to serum albumin ratio. In the 53 observations where the IgG Index is ≤ 1.14, there is a highly significant positive correlation between number of OCBs and the IgG Index (*r* = 0.39, slope = 11.45, se = 3.74, *p* = 0.002); however, in the 16 observations where the IgG Index is greater than 1.14 but less than 1.9, the slope decreases, but not significantly so (*r* = -0.14, slope = -6.09, se = 11.68, *p* = 0.68) (Fig 2C).

The above analyses (Fig 2A–2C) suggest that there are breakpoints in the number of OCBs where important measures in MS behave differentially, suggesting that these relationships are more complex than a simple linear function. We therefore proceeded to categorize OCBs, and performed correlation analysis between OCBs and CSF IgG concentration. In 88 MS patients, the number of OCBs and CSF IgG concentration was significantly correlated (*r* = 0.33, *p* = 0.002). However, in 72% of MS patients when number of OCBs >2, there was no association between number of OCBs and CSF IgG (*r* = 0.12, *p* = 0.35) (Fig 2B and 2D), and the two parameters are correlated when OCBs ≤ 2 (*r* = 0.58, *p* = 0.001, n = 28). The sensitivity analysis revealed that using cut-off values of OCBs > 3 (n = 52) and OCBs > 4 (n = 47), there is a trend towards becoming significantly correlated. At a cut-off value of 5, the p-value reaches 0.04, and the correlation once again becomes increasingly stronger. In 63 MS patients with fewer than 10 OCBs, there is a significant correlation of 0.34 (*p* = 0.006). In the 25 MS patients with OCBs ≥ 10, the linear association between OCBs and CSF IgG was strong and significant (*r* = 0.62, *p* = 0.0009).

### The highly-correlated relationships between serum and CSF proteins in MS

**Fig 3:**
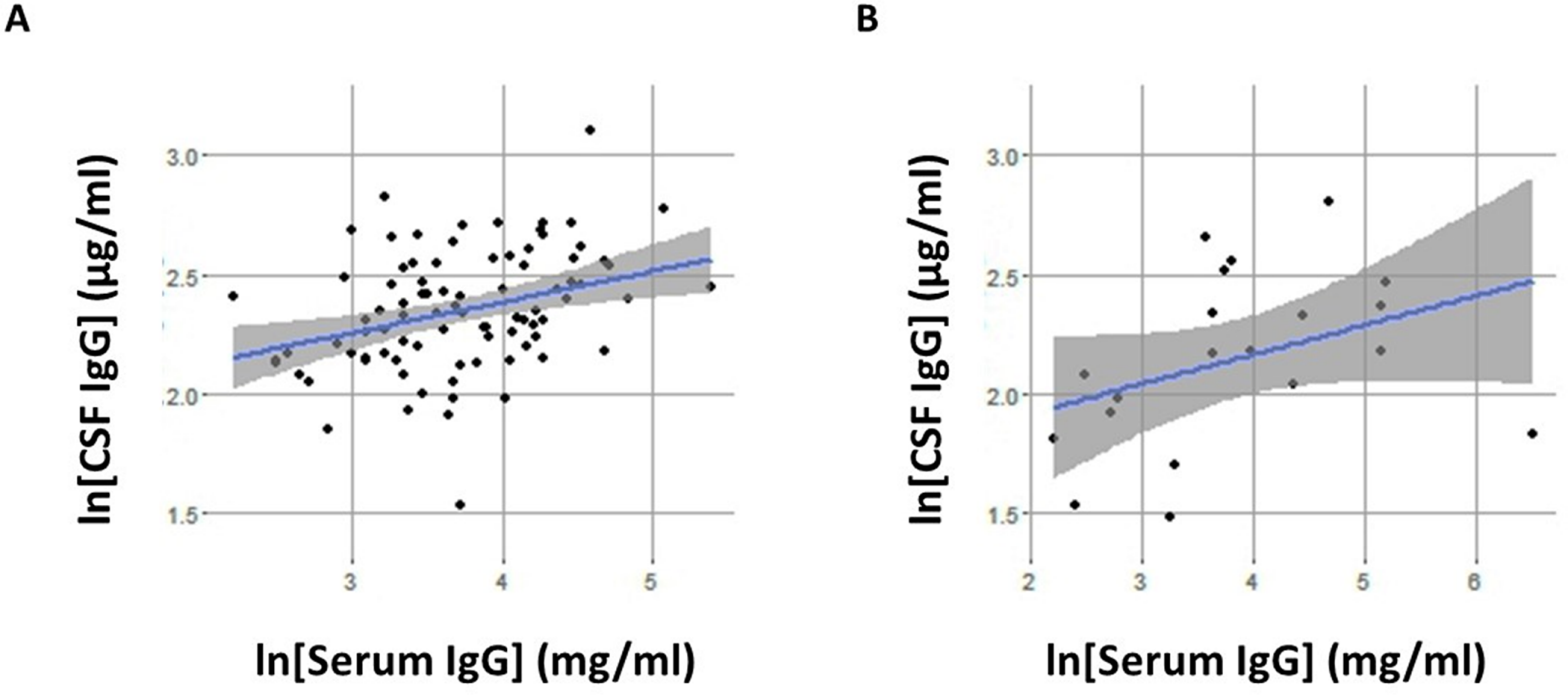
CSF IgG and serum IgG are highly correlated in MS. A. Significant correlation between CSF IgG and serum IgG concentrations is present in MS patients (r = 0.33, p = 0.0015, n = 90); B. In IC patients, CSF IgG is correlated with serum IgG concentrations (r = 0.37, p = 0.1, n = 20).

**Fig 4:**
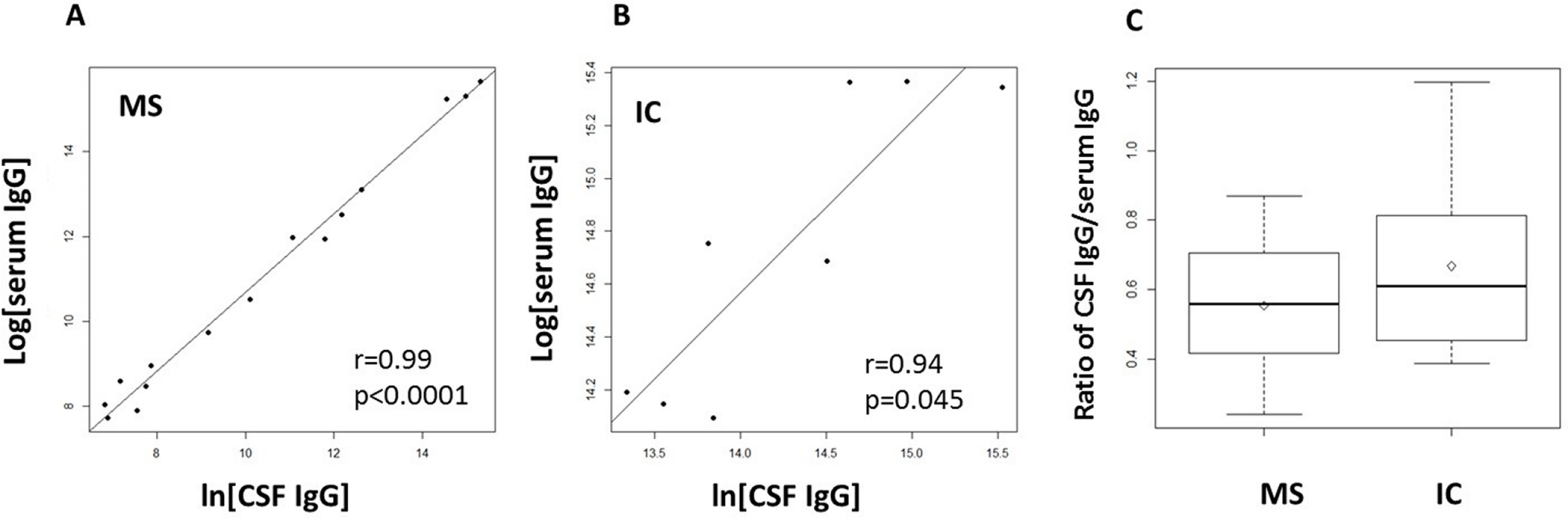
Immunoblot data demonstrate a near perfect correlation between CSF IgG and serum IgG in MS. Slot blot immune-assays with paired CSF and serum were performed with 15 MS and 8 IC. Equal amounts IgG in CSF and sera loaded onto nitrocellulose membranes were probed with anti-IgG (H+L) antibody-HRP followed by chemiluminescent detection. A. A near perfect correlation between CSF IgG and serum IgG concentration is present in MS (r = 0.99, p < 0.0001, n = 15), and in IC (r = 0.88, p = 0.045, n = 8) (B). C. There is no significant difference in the ratio of CSF IgG to serum IgG in MS compared in IC (p = 0.313).

In contrast to the CSF IgG where there is no difference between MS and IC, serum IgG level in MS was significantly elevated (*p* = 0.03). Furthermore, the serum IgG concentration was significantly correlated with the CSF IgG concentration in MS (*r* = 0.33, *p* = 0.0015). To a lesser extent, the serum IgG and CSF IgG was also correlated in IC patients (*r* = 0.37, *p* = 0.10) (Fig 3). The highly-correlated CSF and serum IgG relationships were further demonstrated by slot blots. We demonstrated a near perfect correlation between CSF IgG and serum IgG concentration in MS. However, there is no difference in the ratio of CSF IgG to serum IgG in MS compared to IC (*p* = 0.313) (Fig 4).

**Fig 5:**
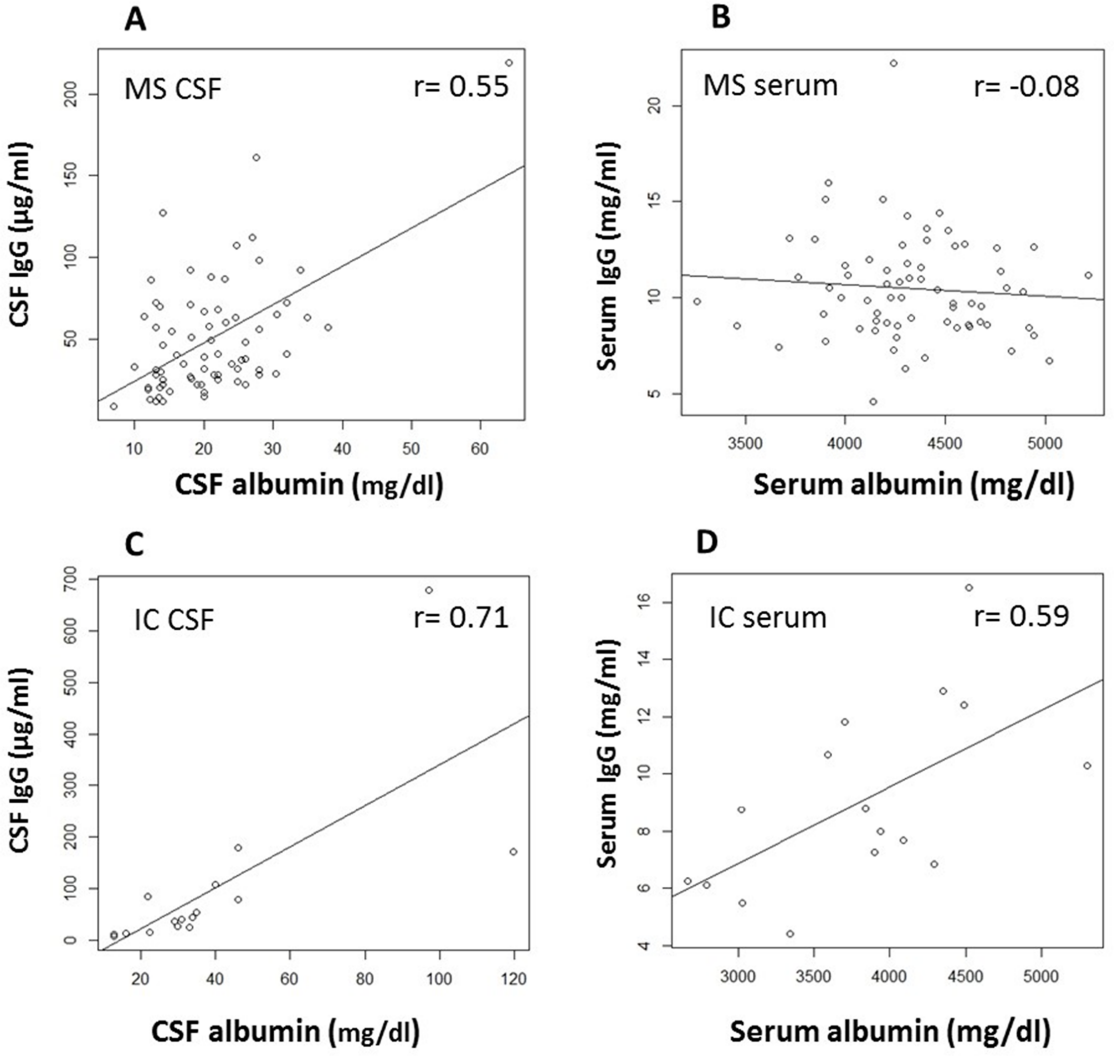
Strong linear correlation between CSF IgG and CSF albumin is found only in MS CSF but not in MS serum. A. Highly significantly correlation exists between concentration of CSF IgG and CSF albumin in MS (r = 0.55, p < 0.0001, n = 70). B. No correlation between serum IgG and serum albumin in MS (r = -0.08, p = 0.53, n = 68). In IC patients, highly correlated relationship was found in both CSF (C) and serum (D).

In MS CSF, there was a strong correlation between CSF IgG and CSF albumin (*r* = 0.55, *p* < 0.0001) (Fig 5A). This robust association was further demonstrated by their near perfect alignment on the diagonal of a quantile-quantile plot, suggesting that the two parameters are interchangeable, and CSF IgG has the same distribution as CSF albumin in MS (data not shown). In contrast, no relationship exists between serum IgG and serum albumin in MS (*r* = -0.08, *p* = 0.53) (Fig 5B). High correlations between IgG and albumin were also observed in both CSF and serum in IC patients (Fig 5C and 5D).

**Fig 6:**
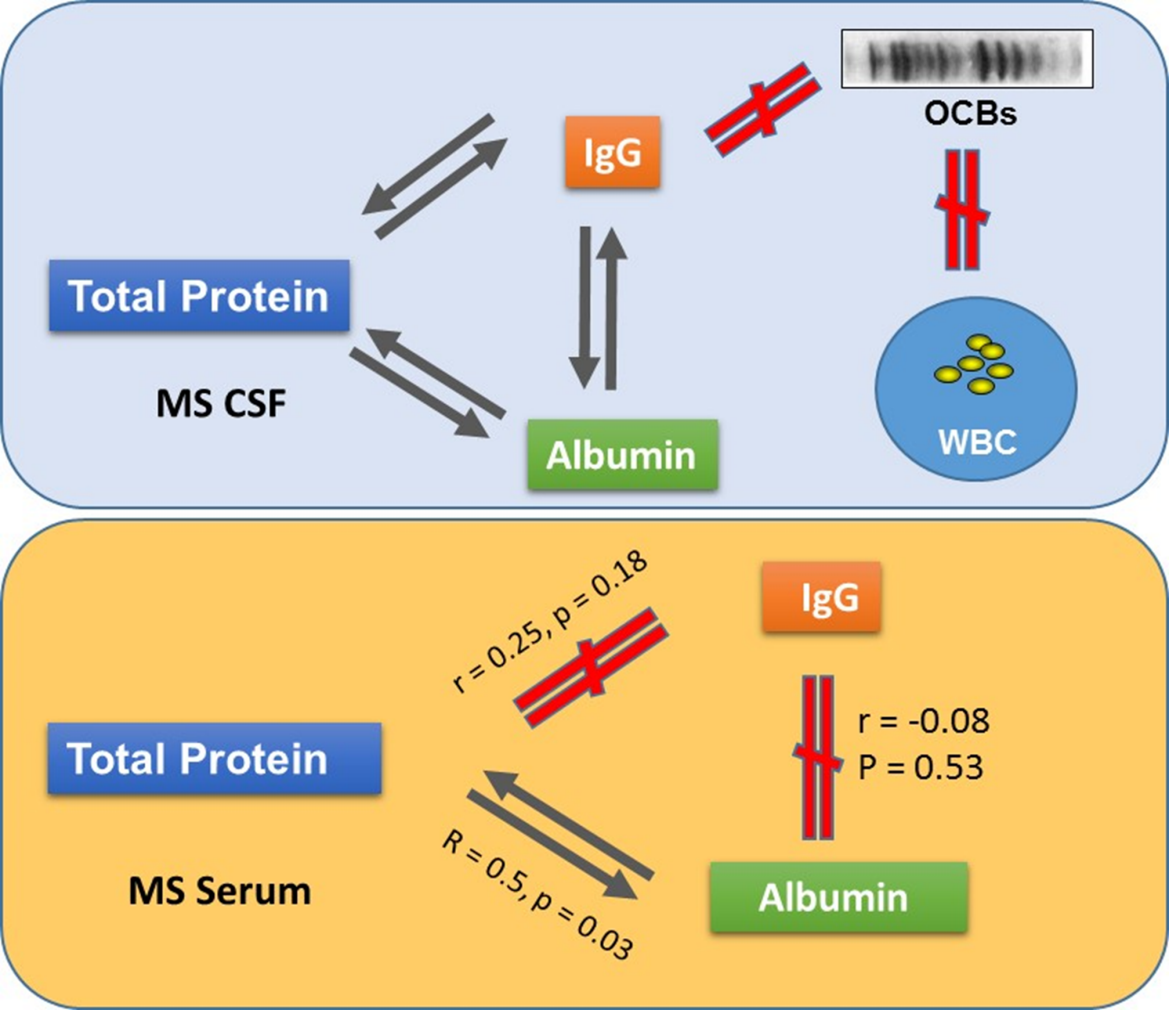
Summary of relationships between the laboratory parameters in CSF and serum of MS. In MS, highly correlated relationships are present between CSF IgG, CSF albumin, and CSF total protein. In contrast, such correlations are not observed in MS serum. Further, there are no direct linear relationship between the number of OCBs, CSF IgG concentration, and CSF cells. Black arrows indicate correlation, and the red ‘not equal’ sign indicates that no correlations were found.

Similarly, in MS there was a strong correlation between the levels of CSF total protein and CSF IgG (*r* = 0.5, *p* = 0.0003), and to CSF albumin (*r* = 0.95, *p* <0.0001). However, no such relationship was found in serum between total protein and IgG, and a lower but still significant correlation was observed between serum albumin and serum total protein (*r* = 0.5, *p* = 0.03) (Table 2). Fig 6 summarizes the relationship between these parameters in MS CSF and serum.

**Table 2:** In MS, there are strong correlation among CSF IgG, CSF albumin, and CSF total protein, but no such relationships are found in serum except between serum albumin and total protein.

**MS CSF**	***r***	***p***	**n**
IgG vs albumin	0.47	p<0.0001	70
IgG vs total protein	0.5	0.0003	48
Albumin vs total protein	0.95	p<0.0001	35
**MS Serum**	***r***	***p***	**n**
IgG vs albumin	-0.08	0.53	68
IgG vs total protein	0.25	0.18	30
ALB vs total protein	0.50	0.03	19

r, correlation coefficient; n, number of data collected.

## Discussion

Elevated intrathecal IgG and OCBs in MS are considered to be an indication of localized B-cell expansion in the brain [[15]], representing the sum of contributions from B cells in the different plaques [[16]]. The high therapeutic efficacy of B cell depletion therapies [[17]] supports the role of B cells in MS. However, our analysis that MS patients have fewer numbers of CSF cells, consistent with findings of others [[14, 18]]. In addition, previous studies have shown that MS CSF contains lower numbers of B cells (5% of CSF cells) compared to both inflammatory and non-inflammatory neurological disorders [[19, 20]].

In MS brains, the presence of IgG containing lymphoid cells is well established [[21]], and ectopic B-cell follicles in the cerebral meninges were detected in 41% of SPMS (but not in PPMS) [[22]]. However, large amounts of IgG representing OCBs can be eluted from plaques in which such cells are absent [[23, 24]], and the highest levels of IgG were observed in old rather than recent plaques, suggesting that IgG can be detected in the vicinity of a plaque for longer than lymphoid cells [[25]]. Our finding that there is no linear relationship between OCBs and CSF cells in MS is consistent with these observations.

The contradiction between the lower number of CNS cells (about 3.5 million cells in MS CSF) and higher levels of intrathecal IgG (10 mg/day), combined with the evidence that there is no correlation between plasma cells and Gd-enhancing lesions in MS, raise the question as to whether B cells in MS CNS can be responsible for the massive amounts of elevated intrathecal IgG. Tourtellotte calculated that it would take 3.2 billion lymphocytes in MS to generate such large amounts of CNS IgG, therefore circulating MS CSF lymphocytes could only account for <0.1% of the extra IgG in the CSF. This apparent knowledge gap suggests that most of the intrathecal IgG in MS may actually be derived from the blood.

It is estimated that 20–40% of the total IgG in MS CSF are contributed to OCBs [[26]]. However, we show that in most of MS patients when OCBs > 2, there is no linear association between CSF IgG and number of OCBs. We further show that there is no linear relationship between number of OCBs and IgG Index, consistent with data as reported by Mares et al., [[27]]. These findings, combined with data that different OCB patterns with various band intensity are present in different regions of the same MS brain, and even from the same plaques [[16, 23, 28]], do not support the theory that one OCB equates with the product of one B cell clone. Further, if B cell clones are recruited randomly to the MS brain then, with time, the OCB spectrum should change whenever a new plaque is formed [[29]]. However, a cardinal feature of MS is that OCBs are very stable over periods of years despite changes in clinical status [[30]]. We therefore propose the hypothesis that each OCB contains multiple IgG species forming IgG complexes.

The presence of a heterogeneous composition of OCBs and IgG immune complexes in MS brain has been suggested by several previous studies. Both IgG1 and IgG3 subclasses are found in the same band, indicating a micro-heterogeneous composition of the OCBs [[31]]. In addition, significant higher amounts of bound IgG were eluted from MS brain with both high and low pH buffer [[23]]. Recent molecular data demonstrated that clonally related and even the same IgG-VH sequences are found in multiple OCB bands [[13]], further support that OCB may represent IgG complexes detectable only in IEF under non-denaturing and non-reducing conditions. The complex relationship between OCBs and other CSF parameters suggests that at certain concentrations, the IgG antibody is being sequestered or aggregated (form IgG complexes) and unable to contribute to the number of OCBs, and the relationships become negatively associated. Once again, these data indicate that the non-linear relationship may be due to the presence of IgG complexes.

The vascular origin of MS has been speculated over a century ago [[32]]. Here we demonstrated a strong correlation between CSF and serum IgG, indicating that the peripheral blood may contribute significantly to intrathecal IgG. We further show that when OCBs ≤ 10, it has a strong association with both CSF IgG (*r* = 0.34, *p* = 0.006) and serum IgG (*r* = 0.27, *p* = 0.03). The similar correlation patterns further suggest a strong link between the two compartments. Our results are in agreement with earlier work that there is an association between high levels of intrathecal synthesis of IgG and serum OCBs [[33]]. Link [[34]] failed to find any immunochemical difference between CSF and serum derived IgG in MS patients, and Tourtellotte et al. [[15]] have shown that serum-derived IgG has the same half-life in CSF as in serum, further supporting the notion that the CSF IgG may have a systemic origin.

The strong correlation between MS CSF IgG and albumin argues that the two parameters are interchangeable, similar as that in healthy controls [[35]], suggesting that CSF may not be the major site contributing pathologically to the disease. Interestingly, no correlation was found between serum albumin and serum IgG in MS, or between albumin in CSF and albumin in serum either. Since CSF albumin is exclusively derived from the blood in MS, and most MS patients have a relatively intact blood-brain-barrier (BBB), the strong correlation of CSF IgG and albumin suggests that most of the CSF IgG is derived from the blood. The most convincing evidence supporting the notion that the serum plays a critical role in CSF IgG generation comes from data that B-cell clusters corresponding to OCBs in MS are observed only in the blood [[13]].

Compelling evidence of serum antibody-mediated demyelination in MS comes from the classic studies of Lumsden [[24]]. They investigated the anti-myelin antibodies in sera from 450 MS patients and found that over 80% of MS sera with natural complement produce demyelination in live cultures of newborn rat cerebellum. Their data support the role of IgG antibodies in their natural state, possibly forming immune complexes with other antibodies/antigens, and are transported across the BBB via binding of the IgG complexes to Fc receptors, resulting in demyelination. It is possible that in MS there exists a combination selective transport, a small BBB leakage, and intrathecal synthesis contributing IgG to the CSF IgG.

In summary, our results suggest that B cells and their products in the CNS may not be the sole source of intrathecal IgG, and OCBs may represent IgG complexes. The peripheral blood may contribute significantly to CSF IgG in MS. The data support a disturbed systemic immunity in MS, and may provide novel opportunities for blood biomarker identification and novel therapeutic targets in MS.
